# Transient Nonlinear Heat Conduction in Concrete Structures: A Semi-Analytical Approach

**DOI:** 10.3390/e25040583

**Published:** 2023-03-29

**Authors:** Hui Wang, Xi Chen, Eduardus Koenders, Ying Dai, Xingchun Huang, Qing Ai, Yong Yuan

**Affiliations:** 1 School of Naval Architecture, Ocean and Civil Engineering, Shanghai Jiao Tong University, Shanghai 200240, China; 2 College of Civil Engineering and Architecture, Jiaxing University, Jiaxing 314001, China; 3 Institute of Construction and Building Materials, Technical University of Darmstadt, 64287 Darmstadt, Germany; 4 School of Aerospace Engineering and Applied Mechanics, Tongji University, Shanghai 200092, China; 5 College of Civil Engineering, Tongji University, Shanghai 200092, China

**Keywords:** concrete heat conduction, semi-analytical approach, high temperature, nonlinear, fire loading

## Abstract

Thermal loading, especially in fire scenarios, challenges the safety and long-term durability of concrete structures. The resulting heat propagation within the structure is governed by the heat conduction equation, which can be difficult to solve analytically because of the nonlinearity related to the thermophysical properties of concrete. A semi-analytical approach for the transient nonlinear heat conduction problem in concrete structures was established in the present work. The nonlinearity related to the temperature-dependent thermal conductivity, mass density, and specific heat capacity of heated concrete was taken into consideration. A Taylor series approximate solution was first established within a small neighborhood, employing the Boltzmann transformation in combination with the mean value theorem. Thereafter, it was extended to the whole domain by utilizing the Bernstein polynomial. The semi-analytical approach was validated by comparing it with the numerical results of two independent Finite Element simulations of nonlinear heat conduction along concrete plates, subjected to either moderate or fierce thermal loading. Absolute values of the relative errors are smaller than 5%. The validated semi-analytical approach was further applied to prediction of the temporal evolution of the temperature field of a scaled model of a subway station, subjected to fire disaster. The nonlinearities, related to the time-dependent surface temperature and the temperature-dependent thermophysical properties of concrete, were taken into consideration. The predictions agree well with the experimental measurements. The established semi-analytical approach exhibits good accuracy and stability, providing insight into the interaction between the thermophysical properties of concrete in the heat conduction process.

## 1. Introduction

Concrete structures are frequently subjected to daily temperature cycles [1,2], sometimes also to extreme weather events such as hail showers [3,4], and occasionally to accidental scenarios such as fire disasters [5,6]. The resulting thermomechanical loading raises not only macroscopic and microscopic thermal stresses [7] but also thermal decomposition of materials [8], which is a challenge to the safety, service performance, and long-term durability of concrete structures.

Identification of the temperature field is the prerequisite for performing a thermomechanical analysis of the behavior of thermally-loaded concrete structures. For example, following the simplified “500 °C isotherm method” recommended by Eurocode 2 [9], concrete with a temperature over 500 °C is ignored, while that with a temperature below 500 °C is considered to maintain its full strength, in quantification of the bearing capacity of concrete members. More sophisticated prediction of the structural performance is related to calibration of the thermal degradation of concrete materials [8,10] and thermal stresses [11,12], as a result of the temporal and spatial evolution of temperature fields. Furthermore, the heat transfer process is also influenced by the mass transfer [13,14], as a result of vaporization and dehydration, and the cracking [15,16], as a result of mechanical response. In this aspect, hygro-thermo-chemo-mechanical models were also proposed [17], which is beyond the scope of this work.

Driven by the temperature difference, heat conducts within concrete, following [18]

(1) $$\rho_{T}\;C_{T}\;\frac{\partial T}{\partial t}=\mathrm{div}\left[K_{T}\;\mathrm{grad}\left(T\right)\right],\;$$

with $\rho_{T}$, $C_{T}$, and $K_{T}$ standing for the mass density, specific heat capacity, and thermal conductivity, respectively. If the partial derivative of temperature with respect to time is nonzero, i.e., ∂T/∂t≠0, the temperature field is time-dependent, defined as transient heat conduction. Stationary heat conduction, in turn, is characterized by a time-independent temperature field, i.e., ∂T/∂t=0. Notably, the effect of mass transfer processes is not explicitly included here for simplicity [19]. The thermophysical properties of concrete are temperature-dependent, leading to the nonlinearity of the governing Equation (1). Both the mass density and the thermal conductivity of concrete decrease with increasing temperature, for example, the empirical functions of these properties recommended by [9]. This is attributed to the water loss [5] as a result of evaporation and dehydration [8] when heated. Apart from temperature, the thermal conductivity of concrete significantly depends on the moisture content, aggregate type, and measurement technique, leading to a fluctuation of experimental results reported in literature [13,20]. Thermal conductivity of concrete at room temperature generally ranges from 1.4W/(m·${}^{\circ }C)$ to 3.6W/(m·${}^{\circ }C)$ [21], which decreases with increasing temperature [9]. Review studies indicate that this thermal conductivity can decrease by around 50% when being heated up to 500 °C [13,22]. The specific heat capacity of concrete at room temperature varies between 840J/(kg·${}^{\circ }C)$ and 1800J/(kg·${}^{\circ }C)$ [5,21], which increases with increasing temperature. This is attributed to the latent heat of the evaporation of free water, the dehydration of cement hydrates, and possible transformation of aggregates [6,21]. Therefore, consideration of the nonlinearity, related to the thermophysical properties of concrete, in a solution of its heat conduction problem, is necessary.

The nonlinear heat conduction problem can be solved either numerically or analytically. An extensive amount of work has been found on the application of Finite Element method in solving the heat conduction problem in concrete structures [23,24], such as pavements [25], tunnel linings [26], and bridge girders [27]. For calibration of the nonlinearity of the partial differential equation, both the mesh size and the time increments shall be set relatively small. Compared to Finite Element simulations, an analytical solution is beneficial in reducing computational efforts and demonstrating the transparent interactions between the thermophysical parameters of concrete in the heat transfer process. The analytical solution is well-known for several linear heat conduction problems [4,28]. Considering the linearity of the governing equation, superposition principles are frequently utilized for the discretization of the time-dependent thermal boundary conditions in construction engineering [29,30]. As for nonlinear heat conduction, various analytical methods have been developed. For example, with Kirchhoff transformation, Kim [31] transferred the steady-state nonlinear heat conduction equation into the Laplace equation, which is easy to solve. By employing the Adomian decomposition method, Arslanturk [32] established an analytical solution in the form of an infinite power series. In the framework of the Differential Transformation Method, the nonlinear differential equation, governing the heat conduction process, can be transferred into algebraic equations in the *K* domain, see [33] for details. Further developments include the homotopy perturbation method [34], the homotopy analysis method [35], the tanh method [36], and so on. Given the mathematical similarity between the governing equations of the heat conduction problem and the diffusion problem, recent progress on solutions of the linear diffusion equation, utilizing three different Ansätze [37], and of the nonlinear diffusion equation, utilizing Boltzmann transformation [38,39], are also worth mentioning. The variable transformation technique of the latter was also followed in the present work.

The present study employed the Boltzmann transformation, in conjunction with the mean value theorem and Bernstein polynomial, for solving transient nonlinear heat conduction of concrete. The nonlinearity related to the temperature-dependent thermophysical properties, i.e., the mass density, specific heat capacity, and thermal conductivity, of concrete and the time-dependent boundary conditions was considered. For establishment of the analytical solution, the Boltzmann variable was first introduced in order to transfer the nonlinear partial differential equation of the heat conduction problem into a nonlinear ordinary differential equation. By utilizing the mean value theorem, an approximate integral form of the governing equation was derived, which was solved by linearization of the nonlinear equation and the first-order Taylor series approximation in a small neighborhood. Finally, the semi-analytical solution for the whole domain was established from the basic solution within the aforementioned neighborhood, by recalling the Bernstein polynomial. The solution was validated by numerical simulations and further applied to a scaled fire test, inspired by a three-span subway station [40]. The thermomechanical behavior of the model in the first 30 min of the test was analyzed by a three-dimensional Finite Element simulation, simulating a non-catastrophic fire event [41]. Notably, the thermophysical properties of concrete were assumed to be constant in simulating the temperature field. This provides the motivation to predict the temperature evolution over the whole heating process, accounting for the temperature-dependent thermophysical properties of concrete.

The present study is structured as follows. Section 2 is devoted to the establishment of the semi-analytical approach of the transient nonlinear heat conduction problem, which was validated by comparing the numerical results with Finite Element simulations of concrete plates in Section 3. The validated semi-analytical approach was further applied to prediction of the temperature evolution of a scaled model of a concrete subway station in Section 4, followed by discussions in Section 5. Conclusions are drawn in Section 6.

## 2. Establishment of the Semi-Analytical Approach

### 2.1. Boltzmann Transformation

In the case of one-dimensional heat conduction, i.e., along the *x*-coordinate, the governing Equation (1) is written as

(2) $$\rho_{T}\;C_{T}\;\frac{\partial T}{\partial t}=\frac{\partial }{\partial x}\left(K_{T}\;\frac{\partial T}{\partial x}\right)\;,$$

where the mass density, specific heat capacity, and thermal conductivity are all considered to be temperature-dependent, i.e., $\rho_{T}=\rho_{T}\left(T\right)$, $C_{T}=C_{T}\left(T\right)$, and $K_{T}=K_{T}\left(T\right)$. An isothermal initial configuration, characterized by a reference temperature of $T_{ref}$, is considered

(3) $$ini.:\;\;T\left(x,t\;=\;0\right)=T_{ref}\;.$$

This reference temperature is considered to remain constant at infinity of a semi-infinite solid, whereas, the interior boundary of this solid is subjected to a temperature change, i.e.,

(4) $$b.c.:\;\;T\left(x\;=\;0,t\right)=T_{hot}\;,\;T\left(x\;=\;+\infty ,t\right)=T_{ref}\;.$$

An intermediate variable, *U*, is introduced as a function of temperature,

(5) $$U=U\left(T\right)=\int_{T_{ref}}^{T}\rho_{T}\;C_{T}\;dT\;,$$

which is defined as the integration of the volumetric heat capacity, i.e., the product of the mass density and specific heat capacity, over temperature. The partial derivatives of *U* with respect to the time *t* and the *x*-coordinate read as

(6) $$\frac{\partial U}{\partial t}=\left(\rho_{T}\;C_{T}\right)\frac{\partial T}{\partial t}\;,\;\frac{\partial U}{\partial x}=\left(\rho_{T}\;C_{T}\right)\frac{\partial T}{\partial x}\;.$$

Substituting Equation (6) into Equation (2) leads to the following expression of the governing equation

(7) $$\frac{\partial U}{\partial t}=\frac{\partial }{\partial x}\;\left(\frac{K_{T}}{\rho_{T}\;C_{T}}\;\frac{\partial U}{\partial x}\right)\;.$$

The initial and boundary conditions are determined by substituting Equations (3) and (4) into Equation (5) as

(8) $$\begin{array}{cc} \; & ini.:\;\;U\left(x,t\;=\;0\right)=U\left(T_{ref}\right)=U_{ref}\;, \end{array}$$

(9) $$\begin{array}{cc} \; & b.c.:\;\;U\left(x\;=\;0,t\right)=U\left(T_{hot}\right)=U_{hot}\;,\;U\left(x\;=\;+\infty ,t\right)=U\left(T_{ref}\right)=U_{ref}\;. \end{array}$$

Following the Boltzmann transformation [38,39,42], the partial differential Equation (7) can be transferred into an ordinary differential equation. A Boltzmann variable ϕ is introduced, as a function of the time *t* and the *x*-coordinate, i.e.,

(10) $$\phi =\frac{x}{\sqrt{t}}\;.$$

The partial derivatives of the Boltzmann variable ϕ with respect to time and *x*-coordinate read as

(11) $$\frac{\partial \phi }{\partial t}=-\frac{\phi }{2\;t}\;,\;\frac{\partial \phi }{\partial x}=\frac{1}{\sqrt{t}}\;.$$

Substituting the Boltzmann variable ϕ into the governing Equation (7) and recalling the implicit differentiation result in

(12) $$\frac{\partial U}{\partial \phi }\;\frac{\partial \phi }{\partial t}=\frac{\partial }{\partial \phi }\;\frac{\partial \phi }{\partial x}\left(\frac{K_{T}}{\rho_{T}\;C_{T}}\;\frac{\partial U}{\partial \phi }\;\frac{\partial \phi }{\partial x}\right)\;,$$

which can be simplified by referring to the partial derivatives of the Boltzmann variable, i.e., Equation (11), as follows:

(13) $$-\frac{1}{2}\;\phi \;\frac{dU}{d\phi }=\frac{d}{d\phi }\left(\frac{K_{T}}{\rho_{T}\;C_{T}}\;\frac{dU}{d\phi }\right)\;.$$

Recalling Equation (5) gives access to the differential, $dU=\rho_{T}\;C_{T}\;dT$, which is then substituted into Equation (13). This leads to the ordinary differential equation, governing the heat conduction process, as

(14) $$-\frac{1}{2}\;\phi \;\frac{\rho_{T}\;C_{T}\;dT}{d\phi }=\frac{d}{d\phi }\left(K_{T}\;\frac{dT}{d\phi }\right)\;.$$

Equation (14) is a transformed version of the heat conduction Equation (2), calibrating the relation between the temperature variable *T* and the Boltzmann variable ϕ. By setting x=0 and x=+∞ in Equation (10), the boundary conditions Equation (4) are transformed to

(15) $$b.c.:\;T\left(\phi \;=\;0,t\right)=T_{hot}\;,\;T\left(\phi \;=\;+\infty ,t\right)=T_{ref}\;.$$

So far, introduction of the Boltzmann variable facilitates the transformation of the nonlinear partial differential equation to a nonlinear ordinary differential equation. An analytical solution of the latter is still challenging, especially when the boundary conditions are time-dependent.

### 2.2. Analytical Solution in a Small Neighborhood

In the following, the nonlinear ordinary differential equation is first solved in a small neighborhood. The governing Equation (14) can be written as

(16) $$-\frac{1}{2}\;\phi \;\rho_{T}\;C_{T}\;dT=d\left(K_{T}\;\frac{dT}{d\phi }\right)\;,$$

with its integration reading as

(17) $$-\frac{1}{2}\;\int_{T_{ref}}^{T}\phi \;\rho_{T}\;C_{T}\;dT=K_{T}\;\frac{dT}{d\phi }\;.$$

By referring to the mean value theorem [43], Equation (17) can be written as

(18) $$-\frac{1}{2}\;\phi \left[T_{ref}+\theta \;\left(T-T_{ref}\right)\right]\;\int_{T_{ref}}^{T}\rho_{T}\;C_{T}\;dT=K_{T}\;\frac{dT}{d\phi }\;,$$

with θ∈(0,1). Taking the integral term on the left-hand-side of Equation (18) to its right-hand-side and integrating the resulting expression leads to

(19) $$-\frac{1}{2}\;\int_{\phi }^{0}\phi \left[T_{ref}+\theta \;\left(T-T_{ref}\right)\right]\;d\phi =\int_{T}^{T_{hot}}\frac{K_{T}}{\int_{T_{ref}}^{T}\rho_{T}\;C_{T}\;dT}\;dT\;.$$

Referring to the mean value theorem for the left-hand-side of Equation (19) again results in

(20) $$\frac{1}{2}\;\phi \left[T_{ref}+\theta \left(T+\vartheta \;\left(T_{hot}-T\right)-T_{ref}\right)\right]\;\phi =\int_{T}^{T_{hot}}\frac{K_{T}}{\int_{T_{ref}}^{T}\rho_{T}\;C_{T}\;dT}\;dT\;,$$

with ϑ∈(0,1). For the convenience of following derivation, a variable ∆T is defined:

(21) $$∆T=T-\left[T_{ref}+\theta \left(T+\vartheta \;\left(T_{hot}-T\right)-T_{ref}\right)\right]\;,$$

such that Equation (20) can be rewritten as

(22) $$\frac{1}{2}\;\phi \left(T-∆T\right)\;\phi \left(T\right)=\int_{T}^{T_{hot}}\frac{K_{T}}{\int_{T_{ref}}^{T}\rho_{T}\;C_{T}\;dT}\;dT\;.$$

A variable $\tilde{T}=T+∆T$ is introduced and substituted into Equation (22), leading to

(23) $$\frac{1}{2}\;\phi \left(\tilde{T}-2\;∆T\right)\;\phi \left(\tilde{T}-∆T\right)=\int_{\tilde{T}-∆T}^{T_{hot}}\frac{K_{T}}{\int_{T_{ref}}^{T}\rho_{T}\;C_{T}\;dT}\;dT\;.$$

Within the small neighborhood of *T*, i.e., ∆T→0, we have the following approximation:

(24) $$\tilde{T}=T+∆T\approx T\;.$$

By substituting Equation (24) into Equation (23), and multiplying the resulting expression with the two sides of Equation (22), respectively, an approximate integral form is derived:

(25) $$\phi \left(T\right)\;\int_{T-∆T}^{T_{hot}}\frac{K_{T}}{\int_{T_{ref}}^{T}\rho_{T}\;C_{T}\;dT}\;dT=\phi \left(T-2\;∆T\right)\;\int_{T}^{T_{hot}}\frac{K_{T}}{\int_{T_{ref}}^{T}\rho_{T}\;C_{T}\;dT}\;dT\;.$$

The approximate governing equation is solved by referring to the first-order Taylor series approximation. ϕ(T) in Equation (25) can be considered as a function of $\left(\int_{T}^{T_{hot}}\frac{K_{T}}{\int_{T_{ref}}^{T}\rho_{T}\;C_{T}\;dT}\;dT\right)$, with its derivative defined as:

(26) $$\phi^{\prime }=\frac{d\phi (T)}{d\left(\int_{T}^{T_{hot}}\frac{K_{T}}{\int_{T_{ref}}^{T}\rho_{T}\;C_{T}\;dT}\;dT\right)}\;.$$

Therefore, the first-order Taylor series approximation of ϕ(T−2∆T) reads as:

(27) $$\phi \left(T-2\;∆T\right)=\phi \left(T\right)+\phi^{\prime }\;\left[\int_{T-2\;∆T}^{T_{hot}}\frac{K_{T}}{\int_{T_{ref}}^{T}\rho_{T}\;C_{T}\;dT}\;dT-\int_{T}^{T_{hot}}\frac{K_{T}}{\int_{T_{ref}}^{T}\rho_{T}\;C_{T}\;dT}\;dT\right]+c\;,$$

with *c* standing for the higher order infinitesimal function, reading as

(28) $$c=O\left({\left[\int_{T-2\;∆T}^{T_{hot}}\frac{K_{T}}{\int_{T_{ref}}^{T}\rho_{T}\;C_{T}\;dT}\;dT-\int_{T}^{T_{hot}}\frac{K_{T}}{\int_{T_{ref}}^{T}\rho_{T}\;C_{T}\;dT}\;dT\right]}^{2}\right)\;.$$

Substituting Equation (27) into Equation (25) results in

(29) $$\begin{array}{cc} \; & \phi \left(T\right)\left(\int_{T-∆T}^{T_{hot}}\frac{K_{T}}{\int_{T_{ref}}^{T}\rho_{T}\;C_{T}\;dT}\;dT-\int_{T}^{T_{hot}}\frac{K_{T}}{\int_{T_{ref}}^{T}\rho_{T}\;C_{T}\;dT}\;dT\right)\\ \; & \;\;=\phi^{\prime }\;\left[\int_{T-2∆T}^{T_{hot}}\frac{K_{T}}{\int_{T_{ref}}^{T}\rho_{T}\;C_{T}\;dT}\;dT-\int_{T}^{T_{hot}}\frac{K_{T}}{\int_{T_{ref}}^{T}\rho_{T}\;C_{T}\;dT}\;dT\right]\;\int_{T}^{T_{hot}}\frac{K_{T}}{\int_{T_{ref}}^{T}\rho_{T}\;C_{T}\;dT}\;dT\\ \; & \;\;\;+c\;\int_{T}^{T_{hot}}\frac{K_{T}}{\int_{T_{ref}}^{T}\rho_{T}\;C_{T}\;dT}\;dT\;. \end{array}$$

For simplicity, two variables, *a* and *b*, are introduced:

(30) $$\begin{array}{cc} a & =\frac{\int_{T-∆T}^{T_{hot}}\frac{K_{T}}{\int_{T_{ref}}^{T}\rho_{T}\;C_{T}\;dT}\;dT-\int_{T}^{T_{hot}}\frac{K_{T}}{\int_{T_{ref}}^{T}\rho_{T}\;C_{T}\;dT}\;dT}{\int_{T-2∆T}^{T_{hot}}\frac{K_{T}}{\int_{T_{ref}}^{T}\rho_{T}\;C_{T}\;dT}\;dT-\int_{T}^{T_{hot}}\frac{K_{T}}{\int_{T_{ref}}^{T}\rho_{T}\;C_{T}\;dT}\;dT}\;, \end{array}$$

(31) $$\begin{array}{cc} b & =\frac{-c}{\int_{T-2\;∆T}^{T_{hot}}\frac{K_{T}}{\int_{T_{ref}}^{T}\rho_{T}\;C_{T}\;dT}\;dT-\int_{T}^{T_{hot}}\frac{K_{T}}{\int_{T_{ref}}^{T}\rho_{T}\;C_{T}\;dT}\;dT}\;, \end{array}$$

Substitution of Equations (30) and (31) into Equation (29) results in

(32) $$\phi =\frac{\phi^{\prime }-b}{a}\int_{T}^{T_{hot}}\frac{K_{T}}{\int_{T_{ref}}^{T}\rho_{T}\;C_{T}\;dT}\;dT\;.$$

This first-order nonlinear ordinary differential equation can be easily solved as

(33) $$\phi =v{\left(\int_{T}^{T_{hot}}\frac{K_{T}}{\int_{T_{ref}}^{T}\rho_{T}\;C_{T}\;dT}\;dT\right)}^{a}+\frac{b}{1-a}\left(\int_{T}^{T_{hot}}\frac{K_{T}}{\int_{T_{ref}}^{T}\rho_{T}\;C_{T}\;dT}\;dT\right)\;,$$

with *v* as a parameter to be determined. At this point, an analytical solution of the nonlinear ordinary differential Equation (14) is established. The latter is transferred to an approximate integral form by utilizing the mean value theorem, which is then solved by recalling the first-order Taylor series approximation. Because of this approximation, the established solution (33) is valid for within the small neighborhood of *T*.

### 2.3. Approximate Solution in the Whole Domain

The approximate solution within the whole domain is established by recalling the Bernstein polynomial [44]. For the sake of distinguishing, the approximate solution in the aforementioned neighborhood, i.e., Equation (33), is denoted as $\phi_{1}$. A variable ξ is introduced as

(34) $$\xi =\frac{\phi_{1}}{\phi_{1,\max}}\;,$$

with $\phi_{1,\max}$ standing for the maxima of $\phi_{1}$ within the neighborhood. Therefore, this variable falls in the range of ξ∈[0,1], by considering its non-negativity. The solution in the whole domain can be considered as a function of the solution within the small neighborhood, that is ϕ=ϕ(ξ). The *n*-th order Bernstein polynomial of ϕ(ξ) follows

(35) $$B_{n}\left(\phi ,n\right)=\sum_{i=0}^{n}\phi \left(\frac{i}{n}\right)\left[\left(\begin{array}{c} n\\ i \end{array}\right)\;\xi^{i}\;{\left(1-\xi \right)}^{n-i}\right]\;,$$

with the binomial coefficient, defined as $\left(\begin{array}{c} n\\ i \end{array}\right)=\frac{n!}{i!\;(n-i)!}$. The Boltzmann variable within the whole domain is approximated by the first-order Bernstein polynomial as

(36) $$\phi \left(\xi \right)\approx B_{n}\left(\phi ,1\right)=\phi \left(0\right)\;\left(1-\xi \right)+\phi \left(1\right)\;\xi \;.$$

At the heated boundary surface, i.e., x=0 and $T=T_{hot}$, the approximate solution in the neighborhood is equal to zero by recalling Equation (33), i.e., $\phi_{1}=0$, and, therefore, the intermediate variable ξ is also equal to zero, i.e., ξ=0. By recalling the definition of the Boltzmann variable in Equation (10), the ϕ(0) in Equation (36) can be quantified as

(37) $$\phi \left(0\right)=\phi \left(\xi =0\right)=\frac{x}{\sqrt{t}}{|}_{x=0}=0\;.$$

Substituting Equations (34) and (37) into Equation (36) results in

(38) $$\phi \left(\xi \right)\approx B_{n}\left(\phi ,1\right)=\frac{\phi (1)}{\phi_{1,\max}}\;\phi_{1}\;,$$

with the fraction standing for a coefficient. Taking Equation (33) into Equation (38) leads to the approximate solution of the Boltzmann variable in the whole domain

(39) $$\phi \approx \frac{v\;\phi (1)}{\phi_{1,max}}{\left(\int_{T}^{T_{hot}}\frac{K_{T}}{\int_{T_{ref}}^{T}\rho_{T}\;C_{T}\;dT}\;dT\right)}^{a}+\frac{b}{1-a}\;\frac{\phi (1)}{\phi_{1,max}}\left(\int_{T}^{T_{hot}}\frac{K_{T}}{\int_{T_{ref}}^{T}\rho_{T}\;C_{T}\;dT}\;dT\right)\;,$$

which can be written as

(40) $$\phi \approx v_{1}{\left(\int_{T}^{T_{hot}}\frac{K_{T}}{\int_{T_{ref}}^{T}\rho_{T}\;C_{T}\;dT}\;dT\right)}^{w}+v_{2}\left(\int_{T}^{T_{hot}}\frac{K_{T}}{\int_{T_{ref}}^{T}\rho_{T}\;C_{T}\;dT}\;dT\right)\;,$$

with parameters $v_{1}$, $v_{2}$, and *w* to be determined.

The boundary condition Equation (15) is recalled for identification of the three parameters. The approximate solution (40) is considered to achieve a minimum residual sum of squares (RSS) of the governing Equation (14) from $T_{ref}$ to $T_{hot}$. Furthermore, the approximate solution accurately satisfy the governing equation at two random temperature points, $T_{1}$ and $T_{2}$, within the boundary scenario $T_{ref}<\left(T_{1}\;,\;T_{2}\right)<T_{hot}$, as

(41) $$\left\{\begin{array}{c} RSS=\int_{T_{ref}}^{T_{hot}}{\left[\frac{1}{2}\;\phi \;\rho_{T}\;C_{T}\;\frac{dT}{d\phi }+\frac{d}{d\phi }\left(K_{T}\;\frac{dT}{d\phi }\right)\right]}^{2}dT\;\;⟶\;\;\min\;,\\ -\frac{1}{2}\;\phi \;\rho_{T}\;C_{T}\;\frac{dT}{d\phi }{|}_{T=T_{1}}\;=\;\frac{d}{d\phi }\left(K_{T}\;\frac{dT}{d\phi }\right){|}_{T=T_{1}}\;,\\ -\frac{1}{2}\;\phi \;\rho_{T}\;C_{T}\;\frac{dT}{d\phi }{|}_{T=T_{2}}\;=\;\frac{d}{d\phi }\left(K_{T}\;\frac{dT}{d\phi }\right){|}_{T=T_{2}}\;. \end{array}\right.$$

The flowchart of the general calculation procedure is summarized as follows:

1. Substitution of the temperature-dependent expressions of the mass density, specific heat capacity, and thermal conductivity, i.e., $\rho_{T}=\rho_{T}\left(T\right)$, $C_{T}=C_{T}\left(T\right)$, and $K_{T}\;=\;K_{T}\left(T\right)$, into the approximate solution of the Boltzmann variable, i.e., Equation (40);
2. Determination of two random temperature points within the boundary scenario, i.e., $T_{ref}<\left(T_{1}\;,\;T_{2}\right)<T_{hot}$, where the approximate solution of the Boltzmann variable (40) accurately satisfies the governing Equation (14), i.e., the last two formulas of Equation (41);
3. Minimization of the residual sum of squares of the result, by taking the approximate solution of the Boltzmann variable (40) into the governing Equation (14), from $T_{ref}$ to $T_{hot}$, i.e., the first formula of Equation (41);
4. Quantification of the three undetermined parameters of the approximate solution (40), i.e., $v_{1}$, $v_{2}$, and *w*, by solving the aforementioned three formulas of Equation (41);
5. Calculation of the temperature field at each time instant by recalling the definition of the Boltzmann variable, i.e., Equation (10).

So far, the semi-analytical approach has been established with consideration of constant boundary conditions. An extension of the approximate approach to time-dependent boundary conditions was developed as follows. The boundary conditions read as

(42) $$b.c.:\;\;T\left(x\;=\;0,t\right)=T_{hot}\left(t\right)\;,\;T\left(x\;=\;+\infty ,t\right)=T_{ref}\;.$$

By referring to the definition of the Boltzmann variable in Equation (10), the *x*-coordinate can be expressed as

(43) $$x=\phi \;\sqrt{t}\;,$$

which can be solved by referring to Equation (40)

(44) $$x=\sqrt{t}\left[v_{1}{\left(\int_{T}^{T_{hot}\left(t\right)}\frac{K_{T}}{\int_{T_{ref}}^{T}\rho_{T}\;C_{T}\;dT}\;dT\right)}^{w}+v_{2}\left(\int_{T}^{T_{hot}\left(t\right)}\frac{K_{T}}{\int_{T_{ref}}^{T}\rho_{T}\;C_{T}\;dT}\;dT\right)\right]\;.$$

Substituting Equation (43) into Equation (2) and recalling the general formula for derivative of implicit function lead to the governing equation as follows:

(45) $$\rho_{T}\;C_{T}\;\frac{\partial x}{\partial t}=-\frac{\partial }{\partial T}\;\left(K_{T}\;\frac{\partial T}{\partial x}\right)\;.$$

For the identification of the three parameters in Equation (44), the approximate solution is considered to achieve a minimum residual sum of squares (RSS) of the governing Equation (45) from $T_{ref}$ to $T_{hot}\left(t\right)$ at a time instant $t_{3}$. Furthermore, the approximate solution accurately satisfies the governing equation for two random temperature points $T_{1}$ and $T_{2}$ at the time instants of $t_{1}$ and $t_{2}$, respectively, as follows:

(46) $$\left\{\begin{array}{c} RSS=\int_{T_{ref}}^{T_{hot}\left(t\right)}{\left[\rho_{T}\;C_{T}\;\frac{\partial x}{\partial t}+\frac{\partial }{\partial T}\left(K_{T}\;\frac{\partial T}{\partial x}\right)\right]}^{2}dT{|}_{t=t_{3}}\;\;⟶\;\;\min\;,\\ \rho_{T}\;C_{T}\;\frac{\partial x}{\partial t}{|}_{t=t_{1},\;T=T_{1}}\;=\;-\frac{\partial }{\partial T}\left(K_{T}\;\frac{\partial T}{\partial x}\right){|}_{t=t_{1},\;T=T_{1}}\;,\\ \rho_{T}\;C_{T}\;\frac{\partial x}{\partial t}{|}_{t=t_{2},\;T=T_{2}}\;=\;-\frac{\partial }{\partial T}\left(K_{T}\;\frac{\partial T}{\partial x}\right){|}_{t=t_{2},\;T=T_{2}}\;. \end{array}\right.$$

The flowchart of the general calculation procedure is summarized as follows:

1. Substitution of the temperature-dependent expressions of the mass density, specific heat capacity, and thermal conductivity, i.e., $\rho_{T}=\rho_{T}\left(T\right)$, $C_{T}=C_{T}\left(T\right)$, and $K_{T}=K_{T}\left(T\right)$, as well as the time-dependent boundary condition $T_{hot}=T_{hot}\left(t\right)$, into the approximate solution of the *x*-coordinate, i.e., Equation (44);
2. Determination of two random temperature points within the boundary scenario at two random time instants, i.e., $T_{ref}<T_{1}<T_{hot}\left(t_{1}\right)$ and $T_{ref}<T_{2}<T_{hot}\left(t_{2}\right)$, where the approximate solution of the *x*-coordinate (44) accurately satisfies the governing Equation (45), i.e., the last two formulas of Equation (46);
3. Minimization of the residual sum of squares of the result at a random time instant, by taking the approximate solution of the *x*-coordinate (44) into the governing Equation (45), from $T_{ref}$ to $T_{hot}\left(t_{3}\right)$, i.e., the first formula of Equation (46);
4. Quantification of the three undetermined parameters of the approximate solution (44), i.e., $v_{1}$, $v_{2}$, and *w*, by solving the aforementioned three formulas of Equation (46);
5. Calculation of the temperature field at each time instant by recalling the solution of the *x*-coordinate, i.e., Equation (44).

## 3. Numerical Study for Validation

**Figure 1:**
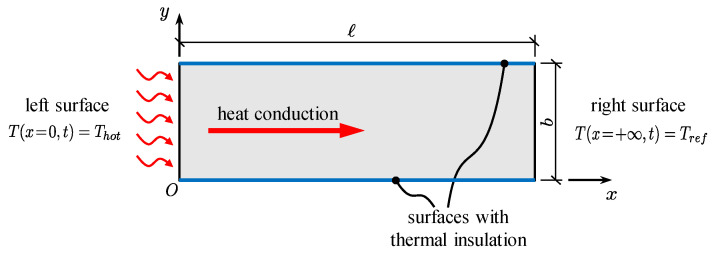
One-dimensional nonlinear heat conduction along the *x*-direction of the plate.

The approximate approach of the nonlinear heat conduction is compared with Finite Element simulations for validation. A concrete plate, characterized by an isothermal initial configuration of temperature $T_{ref}$, was considered. This temperature was assumed to remain constant at the right surface of the plate, whereas the left surface was subjected to a sudden temperature increase to $T_{hot}$ at the time instant: t=0. By considering the other surfaces to be thermally insulated, this leads to one-dimensional heat conduction along the *x*-direction of the plate, see Figure 1.

The numerical simulations consist of two examples. *Example I* refers to a normal concrete plate, subjected to a moderate fire loading below 100 °C, where a temperature-dependent thermal conductivity but a constant specific heat capacity and mass density are considered. *Example II* refers to a self-consolidating concrete plate, subjected to a much more fierce fire loading, reaching the magnitude of 400 °C, where all the three thermophysical parameters are considered to be temperature-dependent. The commercial software COMSOL Multiphysics [45] is used for the Finite Element simulations. The commercial software Maple [46] is used for the numerical implementation of the closed-form solution in this work.

### 3.1. Example I: Normal Concrete Plate, Subjected to a Moderate Fire Below 100 °C

The initial temperature is set equal to $T_{ref}=20\;{}^{\circ}C$ and the sudden temperature increase is set as $T_{hot}=80\;{}^{\circ}C$, representing a moderate fire load [7,41]. As the investigated temperature is below 100 °C, the dehydration process is not stimulated [5,8]. Therefore, both the mass density and the specific heat capacity are considered to be constants for normal concrete, which follows [9] as

(47) $$\rho_{T}=2300\;\mathrm{kg}/m^{3}\;,\;C_{T}=900\;J/\left(\mathrm{kg}\cdot {}^{\circ }C\right)\;,$$

respectively. However, the thermal conductivity is still reported to exhibit a slight decrease even in this moderate fire scenario, following [9]

(48) $$K_{T}=2-0.2451\;\left(\frac{T}{100}\right)+0.0107\;{\left(\frac{T}{100}\right)}^{2}\;\;\;W/\left(m\cdot {}^{\circ }C\right)\;,$$

with *T* standing for temperature in the physical unit of Celsius degree. This results in a nonlinear one-dimensional heat conduction along the length of the plate.

**Figure 2:**
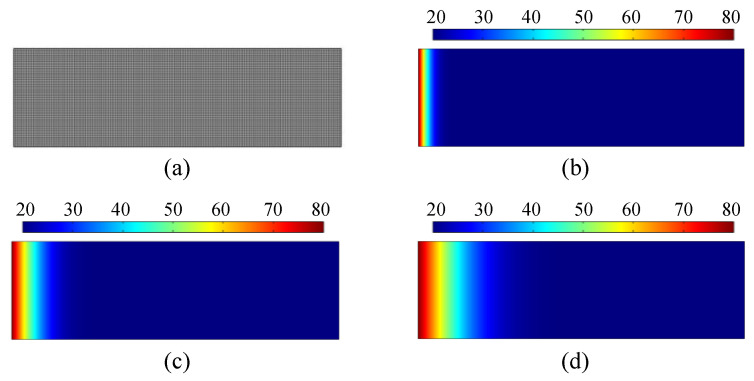
Numerical simulations of the transient nonlinear heat conduction along the length direction of the plate: (**a**) the Finite Element mesh, and the quantified temperature field at time instants of (**b**) 10min, (**c**) 60min, and (**d**) 180min after the thermal shock.

As for the numerical simulation, the length and the width of the plate in Figure 1 are taken as ℓ=1.0m and b=0.3m, respectively. Notably, the width of the plate does not intervene in quantification of the temperature field. The length is set to be long enough in order to satisfy the assumption of the semi-infinite boundary condition, which is validated by the following quantified results. The Finite Element mesh consists of 12,520 quadratic Lagrange elements, which has been checked by a convergence study. The characteristic size of the elements is 0.005m, see Figure 2. The time step size and the tolerance are set equal to 0.01 min and 0.001, respectively.

**Figure 3:**
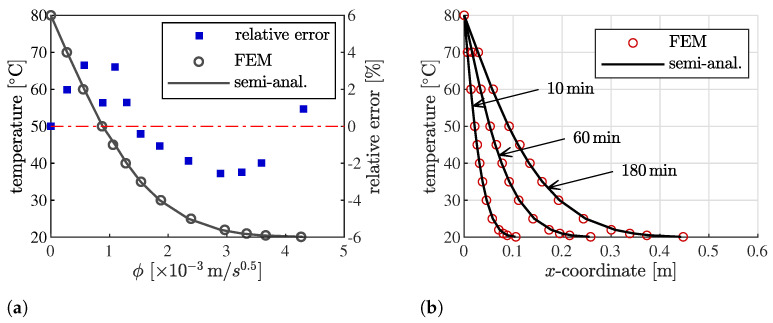
Comparison between the temperature field quantified from the semi-analytical solution and from the Finite Element simulation: (**a**) as a function of the Boltzmann variable $\phi =x/\sqrt{t}$, (**b**) along the length of the plate at the time instants of 10min, 60min, and 180min after the sudden temperature increase, considering a temperature-dependent thermal conductivity while a constant mass density and specific heat capacity.

The numerically quantified temperature field of the concrete plate at the time instant of 10 min, 60 min, and 180 min after the sudden temperature increase is illustrated in Figure 2b,c,d, respectively. Heat progressively propagates from the hot end to the cold end, leading to a growing heated region over time. However, because of the thermal inertia of concrete, the speed of the heat conduction process is rather slow. At the time instant of 180 min after the thermal shock, the temperature of the right half side of the plate still remains constant at 20 °C, see Figure 2d. By recalling the definition of the Boltzmann variable $\phi =x/\sqrt{t}$, the temporally- and spatially-dependent temperature in Figure 2 can be presented as a function of the Boltzmann variable, see the circular markers in Figure 3a. The temperature is nonlinearly distributed along the length of the plate and large temperature gradients are observed in the vicinity of the heated surface.

The semi-analytical solution follows from substituting Equations (47) and (48) into Equation (40). The three parameters $v_{1}$, $v_{2}$, and *w* are identified by solving Equation (41), setting $T_{1}=21\;{}^{\circ}C$ and $T_{2}=50\;{}^{\circ}C$. The latter are random numbers within the range of (20 °C,80 °C). The identified parameters read as

(49) $$v_{1}=-21058.6888\;,\;\;v_{2}=2614.2946\;,\;\;w=1.2010\;.$$

The semi-analytical solutions is compared with the Finite Element simulation and agrees fairly well with the latter, see Figure 3a. The relative error of the semi-analytical solution, by taking results of the numerical simulation as the reference, is quantified as

(50) $$\varepsilon =\frac{T_{ana}-T_{FEM}}{T_{FEM}}\times 100\;\%\;,$$

whose absolute value is found to be smaller than 4%, see the squares in Figure 3a. The distribution of the temperature field along the length of the plate at the time instants of 10min, 60min, and 180min after the sudden temperature increase is shown in Figure 3b, respectively. Within the first 10min, only 0.11m-thick concrete, close to the heated surface, is influenced by the thermal shock. This thickness increases as the heat conducts progressively, until a magnitude of 0.44m at the time instant of 180min after the application of the sudden heating. Therefore, the temperature at the surface x=ℓ is still equal to the reference temperature, satisfying the assumption of semi-infinite boundary condition as in Figure 1.

### 3.2. Example II: Self-Consolidating Concrete Plate, Subjected to a Fierce Fire Reaching 400 °C

In the following exemplary study, the initial temperature was still set equal to $T_{ref}\;=\;20\;{}^{\circ}C$, whereas the sudden temperature increase was taken as $T_{hot}=400\;{}^{\circ}C$, representing a much more fierce fire scenario compared to that of *Example I*. All the three related thermophysical parameters were assumed to be temperature-dependent. For distinguishing with *Example I*, the self-consolidating concrete (SCC) is considered, with its thermal conductivity follows [47],

(51) $$K_{T}=3.12-0.0045\;T\;\;\;W/\left(m\cdot {}^{\circ }C\right)\;,$$

and the product of its mass density and specific heat capacity follows [47],

(52) $$\rho_{T}\;C_{T}=\left(2.4+0.001\;T\right)\times 10^{6}\;\;\;J/\left(m^{3}\cdot {}^{\circ }C\right)\;,$$

where *T* standing for temperature in the physical unit of Celsius degree.

**Figure 4:**
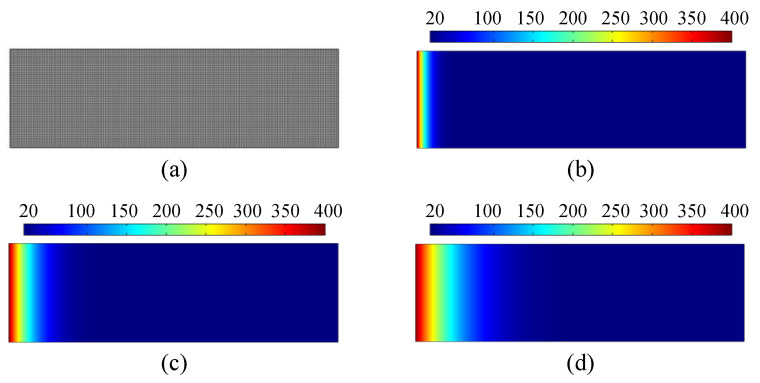
Numerical simulations of the transient nonlinear heat conduction along the length direction of the plate: (**a**) the Finite Element mesh, and the quantified temperature field at time instants of (**b**) 10min, (**c**) 60min, and (**d**) 180min after the thermal shock.

As for the numerical simulation, the geometrical dimensions of the plate and the Finite Element mesh are the same as those of *Example I*. The numerical simulated distribution of the temperature, at the time instant of 10 min, 60 min, and 180 min after the sudden temperature increase is illustrated in Figure 4b,c,d, respectively. Similarly, heat propagates slowly from the hot end to the cold end in this transient scenario. The limited heated region is related to a significant temperature gradient close to the thermally-loaded surface, especially right after the thermal shock, see Figure 4b. The large temperature gradients leads to incompatible thermal eigenstrains, as a product of the temperature change and thermal expansion coefficient, which can result in remarkable thermal stresses [2,4]. This is considered to be one of the main mechanisms of surface spalling of concrete structures, subjected to high temperature [48,49]. The quantified temperature evolution can also be presented as a function of the Boltzmann variable $\phi =x/\sqrt{t}$, see the circular markers in Figure 4a.

Similarly, the semi-analytical solution of the nonlinear heat conduction is established, by substituting Equations (52) and (51) into Equation (40). The three parameters, $v_{1}$, $v_{2}$, and *w*, are identified by solving Equation (41), setting $T_{1}=21\;{}^{\circ}C$ and $T_{2}=197\;{}^{\circ}C$. The latter are random numbers within the range of (20 °C,400 °C). The identified parameters read as

(53) $$\begin{array}{c} v_{1}=-11,824.8806\;,\;\;v_{2}=7213.9428\;,\;\;w=1.0500\;. \end{array}$$

**Figure 5:**
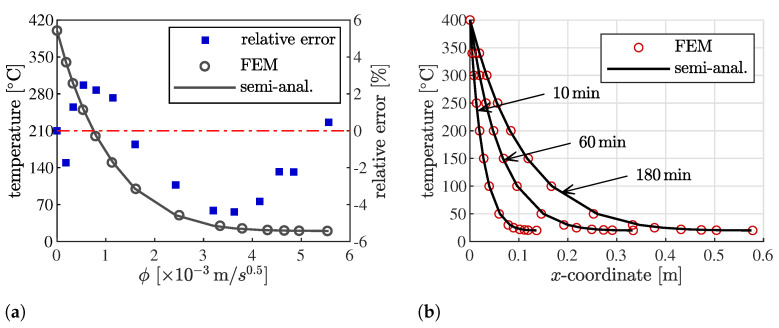
Comparison between the temperature field quantified from the semi-analytical solution and from the Finite Element simulation: (**a**) as a function of the Boltzmann variable $\phi =x/\sqrt{t}$, (**b**) along the length of the plate at the time instants of 10min, 60min, and 180min after the sudden temperature increase, considering a temperature-dependent thermal conductivity, mass density, and specific heat capacity.

A comparison of the semi-analytical solution with the Finite Element simulation is illustrated in Figure 5, which agrees well. The relative errors of the semi-analytical solution, compared to the Finite Element simulation, are quantified by recalling Equation (50), with their maximum absolute values smaller 5%, see Figure 5a. This validates the applicability of the established semi-analytical approach for fierce fire scenario, where the temperature-dependence of all the three thermophysical properties, i.e., the mass density, specific heat capacity, and thermal conductivity, are activated. The temperature distributions along the *x*-coordinate of the plate at the time instant of 10 min, 60 min, and 180 min after the thermal shock are illustrated in Figure 5b. The temperature is nonlinearly distributed along the length of the plate, with significant temperature gradients prevailing in the vicinity of the heated surface. This leads to the mismatch of the expansive thermal eigenstrain of concrete and, thus, compressive stresses in parallel to the heated surface, which may lead to brittle spalling of the surface concrete [48,49]. At the time instant of 10min after the thermal shock, the outermost 0.14m-thick concrete is heated. As heat conduction proceeds, this thickness progressively increases to around 0.58m at time instant of 180min after the application of the sudden heating. The temperature at the far-field boundary keeps constant at the reference temperature.

## 4. Application to a Fire Test

The validated semi-analytical approach of the transient nonlinear heat conduction problem was further utilized for prediction of the temperature evolution of a scaled model of a subway station. The tested model was subjected to a real fire loading scenario, resulting in a time-dependent surface temperature. The setup and the experimental measurements of the fire test will be presented first, followed by comparison with the prediction from the semi-analytical approach.

### 4.1. Experimental Results of a Fire Test of a Subway Station

**Figure 6:**
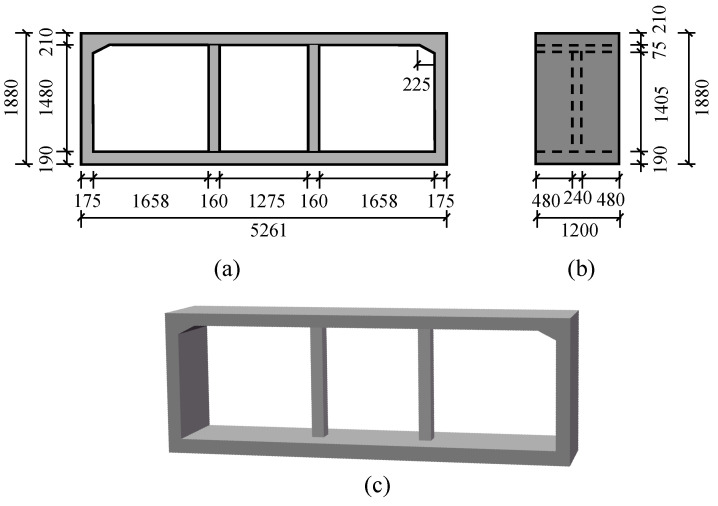
Geometric dimensions of the scaled model of the station hall of a subway station in the fire test: (**a**) front view, (**b**) left view, and (**c**) 3D view (unit: mm), after [40].

The tested structure was stimulated by the station hall layer of a typical subway station in China, in the form of a closed-cell reinforced concrete frame. The tested model represents the substructure at a reduced geometric scale of 1:4, see Figure 6 and details in [40]. Geometrical dimensions of the model read as 5261mm in width, 1880mm in height, and 1200mm in axial length. Its cross section was subdivided into three spans by two rectangular columns in-between. The concrete used for the tested structure was normal concrete of grade C50, in accordance with [50]. The type of the steel bars was Grade HRB400, according to Chinese standard [51].

**Figure 7:**
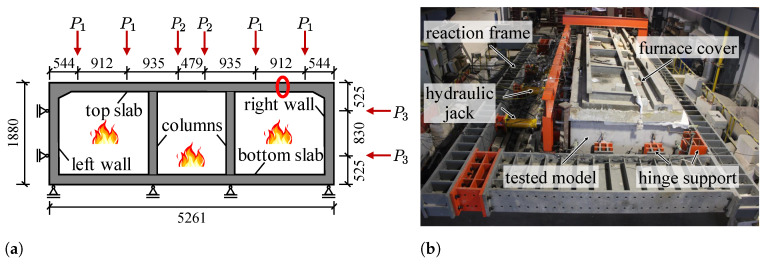
The fire test: (**a**) the mechanical loading and supports (unit: mm) and (**b**) the testing setup, after [40].

**Figure 8:**
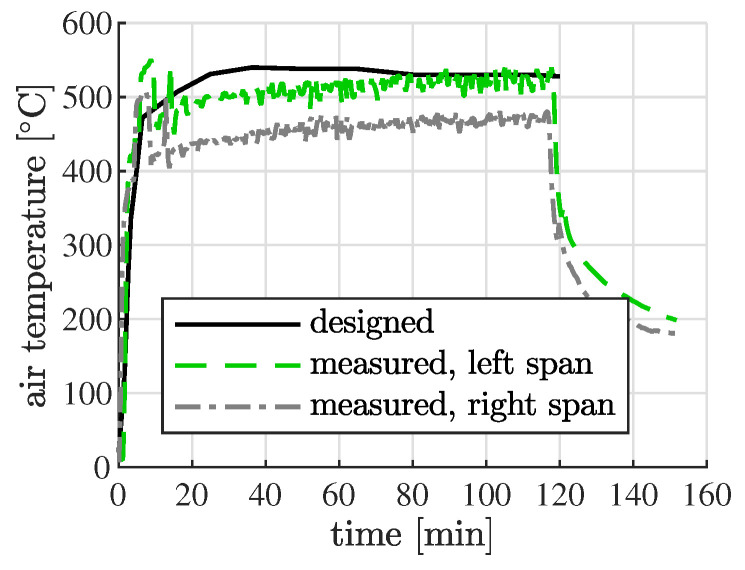
Temperature history of the air in the furnace, applied to the scaled model, after [40].

As for the test setup, the structural model was placed sidelong on the top of a furnace and closed with a fire-resistant cover. Vertical and horizontal earth pressure were simulated by hydraulic jacks acting on the top slab and the right wall, as three sets of concentrated loads. The bottom slab and on the left wall were supported by hinge supports, see Figure 7. The magnitudes of the applied concentrated loads are presented in Table 1. After the application of the mechanical loading, the fire test started. The structure was heated by eight burner nozzles in the furnace, with the temperature of the inside air following the designed temperature history, see the solid graph in Figure 8. The temperature increased quickly to a magnitude of around 500 °C within the first 10min and then kept almost constant till the time instant of 120min. It was followed by natural cooling of the structure by the termination of the applied fire loading. The designed temperature history was determined by simulating the unfavorable fire scenarios in subway station, see [40] for details. The evolution of the air temperature of both the left span and right span of the structure was monitored during the test, which generally agrees well with the designed temperature curve, see Figure 8.

**Figure 9:**
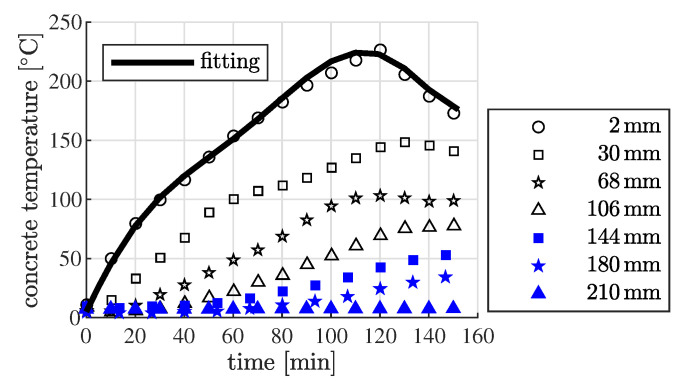
Experimental measured temperature evolution along the thickness at the right span of the top slab, after [40].

Thermocouples were placed at midspan of the three cells of top slab, and at the geometric centers of the bottom slab, of the right column, as well as of the right wall. Six thermocouples were placed equidistantly along the thickness of the right cell of top slab, see the elliptic marker in Figure 7a, in order to monitor the ingress of heat during the fire test. These readings were considered in the following comparison of the semi-analytical solution, as the measurements of temperature along the thickness are integral and detailed [40]. The experimental results are illustrated in Figure 9. The temperature close to the heated inner surface of the top slab increased to a magnitude of 230 °C around 120min after the start of the heating process, which is far smaller than that of the heated air. This is attributed to the natural convection between the air and the structure, as well as the thermal inertia of concrete. With the increase of the distance away from the heated surface, the measured temperature change decreases. The temperature of the outermost surface, i.e., 210mm away from the heated surface, remained almost constant throughout the fire test, see Figure 9. This provides the motivation to take the nonlinear heat conduction along the thickness of the top slab as a semi-infinite problem in the following analysis. Furthermore, one-dimensional heat conduction was found to be predominate in the heat transfer process along the top slab of the tested model [41], which was also adopted in the following analysis.

### 4.2. Prediction of the Temperature Field and Discussion

The tested model of the subway station was stored inside the laboratory before the fire test, leading to an isothermal initial configuration. The initial reference temperature was set equal to $T_{ref}=5\;{}^{\circ}C$, see the measurements at the time instant, t=0min, in Figure 9. As regards the boundary conditions, the evolution of the temperature at the inner surface of the top slab was considered to follow the measurements at a distance of 2mm away from the heated surface, see the circular markers in Figure 9. For the sake of integration and differential in the establishment of the semi-analytical solution, the measurements were fitted by a polynomial as

(54) $$\begin{array}{cc} T_{hot}\left(t\right)= & (1.04699774\times 10^{-20}\;t^{6}-2.44761334\times 10^{-16}\;t^{5}+1.86813256\times 10^{-12}\;t^{4}\\ \; & -4.43751926\times 10^{-9}\;t^{3}-7.65041066\times 10^{-6}\;t^{2}+7.321609883\times 10^{-2}\;t+5){\;}^{\circ }C\;, \end{array}$$

with *t* standing for time in physical units of seconds. It is very similar to the readings of the thermocouple, see the solid graph Figure 9. The temperature at the outer infinity was considered to stay constant at the reference temperature $T_{ref}=5\;{}^{\circ}C$, stimulated by the measurements at 210mm away from the heated surface, see Figure 9.

The thermophysical properties of the normal concrete, consisting the scaled model of the subway station, were considered to follow the Eurocode [9]. The thermal conductivity of normal concrete reads as Equation (48). The temperature-dependent mass density and specific heat capacity read as piecewise functions of temperature, with their product following [9]

(55) $$\rho_{T}\;C_{T}=\left\{\begin{array}{c} 2300\times 900\;J/m^{3}\cdot^{\circ }C\;,\;\;\;\;\;\;\;\;\;\;\;20\leq T<100{\;}^{\circ }C\;,\\ 2300\times \left[900+(T-100)\right]\;J/m^{3}\cdot^{\circ }C\;,\;\;\;\;\;\;\;\;\;\;\;100\leq T<115{\;}^{\circ }C\;,\\ 2300\;\left[1-0.002\left(\frac{T-115}{85}\right)\right]\times \left[900+(T-100)\right]\;J/m^{3}\cdot^{\circ }C\;,\;\;115\leq T<200{\;}^{\circ }C\;,\\ 2300\;\left[0.98-0.003\left(\frac{T-200}{200}\right)\right]\times \left[1000+\frac{T-200}{2}\right]\;J/m^{3}\cdot^{\circ }C\;,\;\;\;200\leq T<400{\;}^{\circ }C\;. \end{array}\right.$$

Given the fact that the steel reinforcements barely influence the heat conduction process [7,52,53], the semi-analytical solution is established based on the aforementioned thermophysical parameters of concrete, rather than separately considering those parameters for concrete and steel.

Given the time-dependent temperature of the inner surface of the top slab, the semi-analytical solution of the nonlinear transient heat conduction along the thickness of the top slab follows from substituting Equations (48) and (55) into Equation (44). The three parameters $v_{1}$, $v_{2}$, and *w* are identified by solving Equation (46) for two random temperature points $T_{1}=5.1{\;}^{\circ }C$ and $T_{2}=15\;{}^{\circ}C$ at the time instants of $t_{1}=t_{2}=152\;\min$, respectively. The time instant $t_{3}$ is also set to be equal 152min for simplicity. The identified parameters read as

(56) $$\begin{array}{c} v_{1}=-370,132.1236\;,\;\;v_{2}=1614.4953\;,\;\;w=1.5000\;. \end{array}$$

**Figure 10:**
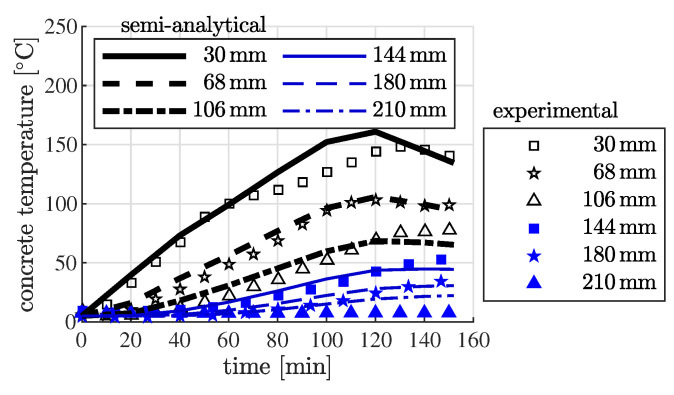
Comparison of the experimentally measured [40] and model predicted temperature evolution during the fire test.

The semi-analytical solution agrees well with the the measured temperature evolution at different positions of the top slab of the heated model, see Figure 10. The maximum temperature difference between the semi-analytical solution and the measurement amounts to 25.7 °C at the position of 30mm away from the heated surface, around 100min after the start of the fire disaster. This can be attributed to the uncertainty of the temperature-dependent thermophysical properties of heated concrete. A more precise prediction of the temperature evolution would require measurements of these properties at high temperatures. The agreement between the semi-analytical solution and the experimental measurements demonstrates that, on the one hand, the applicability of the established semi-analytical solution and, on the other hand, the fact that heat predominantly conducts one-dimensionally along the thickness of the heated top slab in the investigated fire scenario.

## 5. Discussion

### 5.1. Comparison with the Results Obtained by Linear Heat Conduction

From the point of view of practical engineering, the analytical solution of linear heat conduction is frequently used [4,54]. Sorgner et al. [54] presented an engineering mechanics analysis of the subway station in the first 30 min of the aforementioned test, standing for a moderate fire scenario. By considering constant thermophysical properties of concrete, the superposition principle was applied to the resulting linear heat conduction problem, in order to quantify the temperature field. Therefore, the continuous temperature evolution of the heated surface of the top slab is discretized in step-wise fashion, referring to a temperature increment $∆T_{hot,k}=T_{hot}\left(t_{k}\right)-T_{ref}$ at time instant $t_{k}$ ($k=1,2,\ldots ,N_{i}$). Solution of the temperature evolution, resulting from an individual temperature increment, is straightforward and summation of these elementary solutions gives access to the temperature field of the top slab as [54]

(57) $$\begin{array}{cc} T\left(x,t\right)=T_{ref} & +\sum_{k=1}^{N_{i}}∆T_{hot,k}\left(\frac{1}{2}+\frac{z}{h}\right)\\ \; & +\sum_{n=1}^{\infty }\frac{∆T_{hot,k}\;{\left(-1\right)}^{n}}{n\;\pi }\sin\left(2\;n\;\pi \frac{z}{h}\right)\exp\left[-{\left(2\;n\;\pi \right)}^{2}\frac{a\langle t-t_{i}\rangle }{h^{2}}\right]\\ \; & +\sum_{n=1}^{\infty }\frac{∆T_{hot,k}\;{\left(-1\right)}^{n}}{(2n-1)\;\pi }\cos\left[\left(2n-1\right)\;\pi \frac{z}{h}\right]\exp\left[-{\left(2n-1\right)}^{2}\pi^{2}\frac{a\langle t-t_{i}\rangle }{h^{2}}\right]\;, \end{array}$$

where 〈·〉 stands for the Macaulay operator. Notably, the origin of coordinates is set at the middle of the plate for this analytical solution.

**Figure 11:**
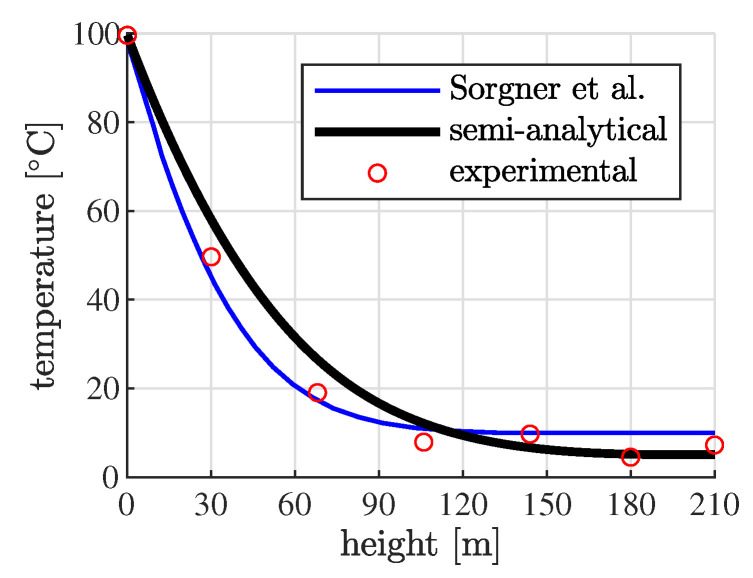
Comparison of the temperature fields obtained by Sorgner et al. [54] for linear heat conduction and by the semi-analytical approach for heat conduction.

The temperature fields of the top slab, obtained from the engineering mechanics analysis [54] and the semi-analytical approach, were compared, see Figure 11 for the results at the time instant of 30 min after the start of the fire. Generally, the quantified temperature fields are quite similar and agree well with the experimental measurements, see the circular markers in Figure 11. The former analysis was conducted by assuming constant thermophysical properties of concrete, whereas their temperature-dependence is considered in application of the semi-analytical approach. As regards the time-dependent boundary condition, the continuous temperature history is fitted by a polynomial and directly substituted into Equation (44) in the semi-analytical approach, instead of discretization. The similarity of the presented results also indicates that the temperature-dependence of the thermophysical properties is of minor importance for the investigated moderate fire scenario, where the temperatures of concrete at all positions are virtually smaller than 100 °C. However, the nonlinearity of the heat conduction problem, related to the temperature-dependent thermophysical properties of concrete, shall be addressed in case of fierce fire scenarios. This is discussed in the following.

### 5.2. Discussion on the Nonlinearity Related to the Thermophysical Properties

The influence of the nonlinearity related to the thermophysical properties of concrete on the heat conduction process is discussed in the following. Both the initial and boundary conditions follow those of *Example II*, that is $T_{ref}=20\;{}^{\circ}C$ and $T_{hot}=400\;{}^{\circ}C$. Three sets, the mass density $\rho_{T}$, specific heat capacity $C_{T}$, and thermal conductivity $K_{T}$, of concrete were investigated.

- S-I: All three thermophysical parameters were considered to be temperature-dependent, following those of *Example II*, i.e., Equations (51) and (52).
- S-II: The thermal conductivity was considered to be temperature-dependent, following Equation (51), whereas both the mass density and the specific heat capacity were considered to be constant, with their product equal to the value of Equation (52) by setting $T=20{\;}^{\circ }C$, i.e., $\rho_{T}\;C_{T}=2.42\times 10^{6}\;J/\left(m^{3}\cdot {}^{\circ }C\right)$.
- S-III: All three thermophysical properties were considered to be temperature-independent, reading as the values of Equations (51) and (52) by setting $T=20{\;}^{\circ }C$, i.e., $K_{T}=3.03\;W/\left(m\cdot {}^{\circ }C\right)$ and $\rho_{T}\;C_{T}=2.42\times 10^{6}\;J/\left(m^{3}\cdot {}^{\circ }C\right)$.

**Figure 12:**
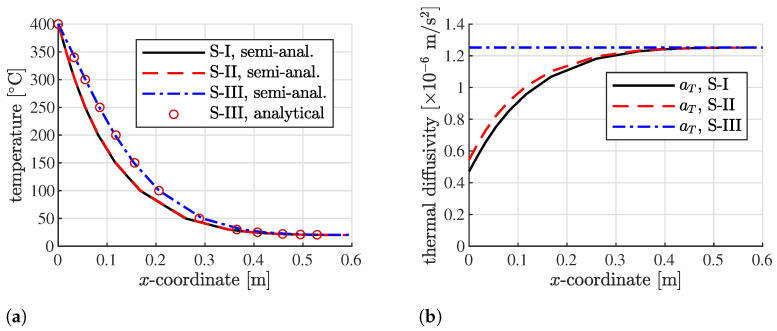
(**a**) The temperature distribution and (**b**) the corresponding thermal diffusivity along the length of the plate at the time instant of 180min, considering temperature-dependent, partly temperature-dependent, and temperature-independent thermophysical properties of concrete.

The semi-analytical solution of the heat conduction, considering thermophysical properties of S-II and S-III, was established, following the aforementioned strategy. Generally, heat propagates similarly along the length of the plate, whereas the speed of the conduction process differs. Temperature distributions along the length of the plate at the time instant of 180min for the three investigated scenarios are illustrated as Figure 12a. Apparently, heat intrudes faster in case of S-III, compared to that of S-I and S-II. For example, at the time instant of 180 min, the magnitude of 100 °C is reached at the depth of 0.1675 m, 0.1685 m, and 0.2062 m for S-I, S-II, and S-III, respectively; the magnitude of 200 °C is reached at the depth of 0.0817m, 0.0825m, and 0.1163m for S-I, S-II, and S-III, respectively. This is attributed to the temperature-independence of the thermal conductivity in case of S-III. The latter stays constantly equal to 3.03W/(m·°C), whereas the ones for S-I and S-II decreases progressively with increasing temperature, following Equation (51). However, the influence of the temperature-dependence of the mass density and the specific heat capacity is of minor importance; see the comparison of the solid and dashed graphs in Figure 12a. Finally, it is worth mentioning that analytical solution is available in case of linear transient heat conduction, i.e., S-III, reading as [55,56]

(58) $$\frac{T\left(x,t\right)-T_{hot}}{T_{ref}-T_{hot}}=\frac{2}{\sqrt{\pi }}\int_{0}^{z}\exp^{-\eta^{2}}\;d\eta \;,$$

with its right hand side standing for the error function of $z=x/\sqrt{4\frac{K_{T}}{\rho_{T}C_{T}}t}$. This classical analytical solution agrees well with the presented semi-analytical solution, see the circular markers in Figure 12a, which further validates the established solution.

For clarification of the nonlinearity related to the thermophysical parameters, the corresponding profile of thermal diffusivity $a_{T}$ at the time instant of 180min is illustrated as Figure 12b. It is quantified as the thermal conductivity divided by the product of the mass density and the specific heat capacity

(59) $$a_{T}=\frac{K_{T}}{\rho_{T}\;C_{T}}\;.$$

The thermal diffusivity stands for the rate at which heat can transfer from the hot end to the cold end within the concrete plate. In the case of S-III, the thermal diffusivity remains constant as $1.25\times 10^{-6}\;m^{2}/s$ along the *x*-coordinate, as a result of the temperature-independent mass density, specific heat capacity, and thermal conductivity of concrete. However, it varies significantly in the case of S-I and S-II, reading as $0.47\times 10^{-6}\;m^{2}/s$ and $0.55\times 10^{-6}\;m^{2}/s$, respectively, at the heated surface with the temperature of concrete as 400 °C and equally as $1.25\times 10^{-6}\;m^{2}/s$ at the far-field boundary with the temperature of concrete as 20 °C, see Figure 12b. This leads to the faster intrusion of heat for S-III and, therefore, a higher temperature, compared to that of S-I and S-II. Decrease of the thermal diffusivity of concrete at high temperature is attributed to the decrease of thermal conductivity, as well as the increase of the mass density and specific heat capacity of concrete. The former contribution dominates, which results in the similar thermal diffusivity in the case of S-I and S-II, see the solid and dashed graphs in Figure 12b.

## 6. Conclusions

In the present paper, a semi-analytical approach to transient nonlinear heat conduction in a concrete structure, subjected to a time-dependent/independent thermal boundary condition, was established. Following the Boltzmann transformation of the partial differential equation to ordinary differential equation, the mean value theorem and Taylor series approximation were used for the solution in a small neighborhood, which was extended to the whole domain by utilizing the Bernstein polynomial. The established semi-analytical approach was first validated by comparing the numerical results with Finite Element simulations and then applied to a fire test. In this respect, the following conclusions are drawn:

- The nonlinearity, related to the temperature-dependent mass density, specific heat capacity, and thermal conductivity of concrete, was taken into consideration. The semi-analytical approach was validated by comparison with two independent Finite Element simulations of heated plates, consisting of normal and self-consolidating concrete, respectively. Absolute values of the difference of the semi-analytical solution, with respect to the numerical results, are smaller than 5% in both exemplary studies.
- By referring to the definition of the Boltzmann variable, the semi-analytical solution was further extended to consideration of a time-dependent thermal boundary condition, which is commonly encountered during the service life of concrete structures. The extended solution was compared with the experimental measurements of a fire test of a scaled concrete subway station. Satisfactory agreement was achieved.

Therefore, the established approach demonstrates its accuracy and stability in solving the transient nonlinear heat conduction problem in concrete structures. From the quantified temperature fields of the investigated scenarios, the following conclusions were drawn:

- Because of the rather small thermal conductivity, concrete generally exhibits good thermal insulation performance. Therefore, the established semi-analytical approach of the nonlinear heat conduction for a *semi-infinite* plate can still be widely used in real scenarios, such as the presented examples of the concrete plates and slabs in a subway station, subjected to either moderate or fierce fire loading.
- The thermal insulation property also leads to significant temperature gradients in the vicinity of the heated surface and, therefore, strong nonlinearity of the thermal eigenstrain, as a product of the temperature difference and the thermal expansion coefficient of concrete. This nonlinearity governs the resulting thermal stresses of concrete structures [4,30]. In the case of a fire test of the scaled model of a subway station, tensile cracking can occur at the inaccessible exterior surface, which is a threat to the long-term durability.

As for the maintenance of concrete structures exposed to potential fire loading, the following recommendations can be made from the present study:

- It is recommended to position enough thermocouples close to the fire-exposed surface. With the knowledge of the temperature evolution of the heated surface, the established semi-analytical approach provides access to the evolution of the temperature fields within concrete structures, serving as the basis for the following thermomechanical analysis and damage evaluation.
- It is recommended to carry out careful inspection of the thermally-loaded concrete structures. The strong nonlinearity of the temperature field is very likely to result in significant thermal stresses and potential thermal cracking.

Finally, it is worth mentioning that the presented semi-analytical approach was established by assuming semi-infinite boundary conditions, which is applicable to some heating scenarios because of the thermal inertia of concrete; for example, the presented fire test with a heating duration of 2 h and a maximum air temperature of around 500 °C. However, in the case of even more fierce fire disasters, for example the Channel Tunnel fire with a heating duration of about 10 h and a maximum air temperature up to 700 °C [57], extension of the present work to finite boundary conditions is required. Moreover, the coupled-effect of mass transfer processes, including the latent heat of vaporization and dehydration, shall also be incorporated in future development of the semi-analytical approach.

## Figures and Tables

**Table 1:** Magnitude of the mechanical loading, applied to the scaled model.

Loading	$P_{1}$	$P_{2}$	$P_{3}$
magnitude [kN]	192.0	151.2	120.0

## Data Availability

Data are available upon request to the authors.
